# Intrinsic Effects of Exposome in Atopic Dermatitis: Genomics, Epigenomics and Regulatory Layers

**DOI:** 10.3390/jcm12124000

**Published:** 2023-06-12

**Authors:** Katerina Grafanaki, Charalabos Antonatos, Alexandros Maniatis, Antonia Petropoulou, Eleftheria Vryzaki, Yiannis Vasilopoulos, Sophia Georgiou, Stamatis Gregoriou

**Affiliations:** 1 Department of Dermatology-Venereology, School of Medicine, University of Patras, 26504 Patras, Greecesgeo@upatras.gr (S.G.); 2 Department of Biochemistry, School of Medicine, University of Patras, 26504 Patras, Greece; 3 Laboratory of Genetics, Section of Genetics, Cell Biology and Development, Department of Biology, University of Patras, 26504 Patras, Greece; charisantonatos@gmail.com (C.A.); iovasilop@upatras.gr (Y.V.); 4 Department of Dermatology-Venereology, Faculty of Medicine, Andreas Sygros Hospital, National and Kapodistrian University of Athens, 16121 Athens, Greece

**Keywords:** intrinsic exposome, atopic dermatitis, miroRNAs, interactome, pregnancy exposome

## Abstract

Atopic dermatitis (AD) or atopic eczema is an increasingly manifested inflammatory skin disorder of complex etiology which is modulated by both extrinsic and intrinsic factors. The exposome includes a person’s lifetime exposures and their effects. We recently reviewed the extrinsic exposome’s environmental risk factors that contribute to AD. The periods of pregnancy, infancy, and teenage years are recognized as crucial stages in the formation of AD, where the exposome leads to enduring impacts on the immune system. However, research is now focusing on the interactions between intrinsic pathways that are modulated by the extrinsic exposome, including genetic variation, epigenetic modifications, and signals, such as diet, stress, and microbiome interactions. As a result, immune dysregulation, barrier dysfunction, hormonal fluctuations, and skin microbiome dysbiosis are important factors contributing to AD development, and their in-depth understanding is crucial not only for AD treatment but also for similar inflammatory disorders.

## 1. Introduction

**Figure 1:**
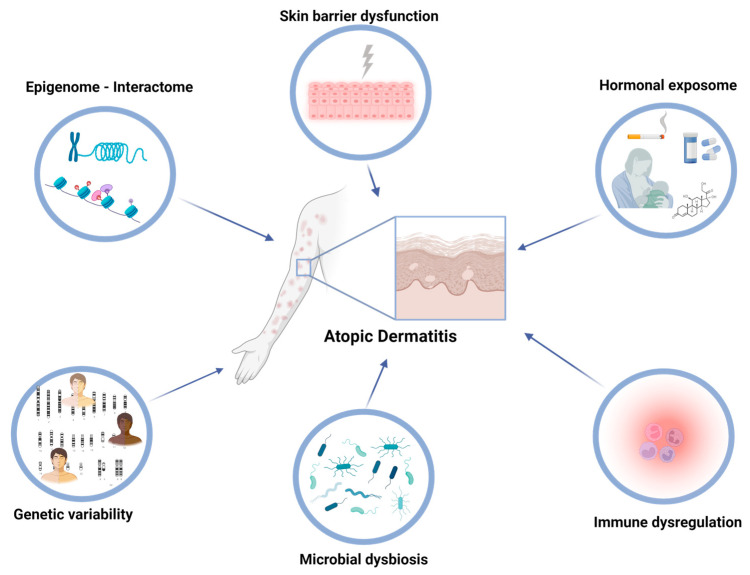
In atopic dermatitis, immune dysregulation, barrier dysfunction, hormonal fluctuations, and alterations in the skin microbiome are key intrinsic factors that can be influenced by both genomic, epigenomic, and environmental factors.

**Figure 2:**
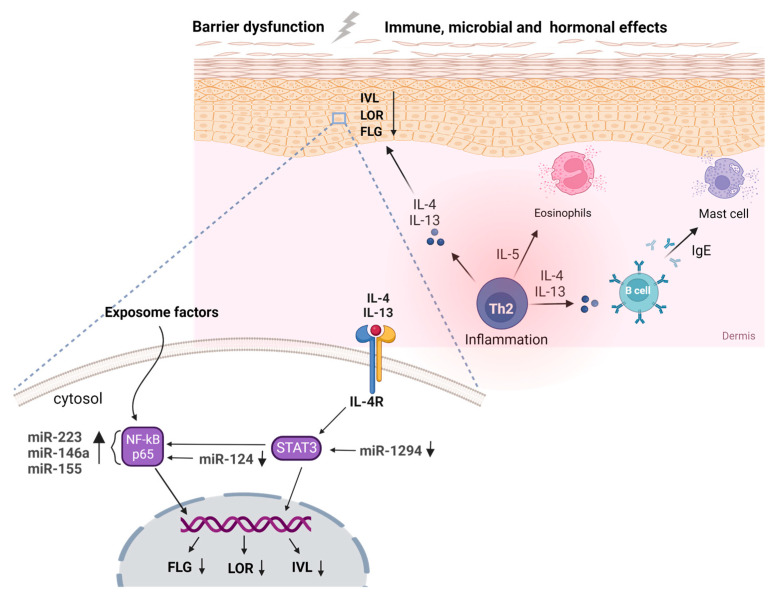
Genetic variations in immune-related genes, specifically those encoding cytokines, such as IL-4 and IL-13, have been linked to a higher susceptibility to atopic dermatitis (AD). Additionally, mutations in the filaggrin gene and other genes responsible for maintaining the integrity of the skin barrier can also contribute to the development of AD. Skin microbiome changes, including a decrease in microbial diversity and an increase in *Staphylococcus aureus* colonization, have also been observed and can be influenced by genetic and environmental factors. Epigenetic modifications to genes involved in these processes and microRNAs, such as miR-1294 and miR-124 via STAT3 and NF-κΒ, in a pivotal role, can further contribute to the development and progression of AD. (Created by Biorender.com (accessed on 10 May 2023)).

Atopic dermatitis (AD), alternatively referred to as atopic eczema, is an inflammatory skin condition that affects approximately 10–20% of children and 1–3% of adults globally, with a growing occurrence observed in developed nations [1]. The exposome refers to the total lifetime exposures of individuals and their corresponding effects. Our recent review comprehensively explored the multifaceted extrinsic exposome, incorporating environmental risk factors and mechanisms that contribute to AD [2]. However, there is growing research interest in understanding the interactions between both intrinsic and extrinsic exposomes in driving AD pathogenesis and maintenance. The intrinsic exposome refers to endogenous factors that contribute to the dysregulated inflammatory and skin barrier pathways in AD (Figure 1). Genetic variability in AD patients holds a crucial role in the disease predisposition, presenting several future opportunities for utilization as disease biomarkers [3,4]. Εxtensive research through candidate-gene approaches and genome-wide association studies (GWASs) has identified specific single nucleotide polymorphisms (SNPs) associated with AD, including genes involved in skin barrier function, immune response, and inflammation. The major example of AD-associated locus refers to the filaggrin (*FLG*) gene, located in the epidermal differentiation complex (EDC); loss-of-function (LOF) variants in *FLG* have been implicated in the impairment of skin barrier integrity, as revealed by candidate-gene approaches, and subsequently validated through GWASs [5]. Additionally, other genes encoding cytokines involved in type 2 inflammation and immune regulation, with major examples including interleukin (IL) IL-4, IL-13, and IL-31, have been linked with the pathogenesis of AD and revealed as risk loci [6] (Figure 2).

Apart from genetic variations, the occurrence of epigenetic modifications and disrupted expression of regulatory molecules have emerged as crucial factors governing gene expression and have been associated with the development of AD. Epigenetic modifications include reversible changes that modulate the transcriptional activity in the absence of changes in the underlying DNA sequence, such as DNA methylation and histone modifications [7]. For instance, aberrant DNA methylation patterns of genes involved in immune response and skin barrier function have been detected in patients with AD, suggesting a potential role for epigenetic modifications in the molecular mechanisms underlying the disease. Furthermore, noncoding RNAs (ncRNAs) have emerged as pivotal regulators of gene expression, and their implication in the pathogenesis of AD has gathered significant attention. The expanding class of ncRNAs incorporates several diverse molecules, such as microRNAs (miRNAs), long noncoding RNAs (lncRNAs), and circular RNAs (circRNAs), with significant discrepancies in both size and related functional roles [8]. Through specific interactions with target genes, these ncRNAs modulate gene expression, exerting profound effects on cellular processes, including immune response, inflammation, and skin barrier function. The perturbed expression of such regulatory molecules has been documented in eczema patients, underscoring their potential role in disease initiation and progression.

The complexity of the clinical exacerbation of eczema, however, derives from the underlying molecular mechanisms that orchestrate the inflammatory response and skin barrier dysfunction. Genetic variants can impact the epigenetic modifications modulating the transcriptional landscape of AD, which in turn can affect the expression of ncRNAs with a predominant regulatory role in the abundance of trans-cellular processes that dynamically interact with exhibiting the pathological condition. Moreover, ncRNAs can also interact with epigenetic machinery to modulate gene expression patterns and contribute to the development of AD. The interplay between intrinsic exposomes is mediated via multi-layered interactions between the genomic, epigenomic, and transcriptional profiles as well as through several factors of the extrinsic exposome, with the microbiome and prenatal effects as major contributors [2]. These complex interactions highlight the convoluted nature of the intrinsic exposome in atopic dermatitis and the need for further research to fully understand these mechanisms. Despite the progress made in understanding the genetics, epigenetics, and ncRNAs of the intrinsic exposome in AD, there are still several challenges that need to be addressed. Understanding the complexity of these factors has important implications for the diagnosis, prevention, and treatment of eczema. In this review, we will delve into the current knowledge on the genetics, epigenetics, and ncRNA regulatory interactions in AD, with the aim to delineate the perturbed molecular profile of eczema patients and illustrate the intricate interplay between genetic and environmental factors in the development of this complex skin disorder.

## 2. Intrinsic Exposome of AD Overview

The intrinsic exposome encompasses endogenous factors that impact a person’s vulnerability to disease, including genetic variation, epigenetic modifications, and cellular processes that can be influenced by environmental factors such as diet, stress, and microbiome. Intrinsic factors, such as immune dysregulation, barrier dysfunction, and alterations in the skin microbiome, play a vital role in the onset and progression of AD. Genetic and environmental factors can affect these intrinsic factors. Understanding these intrinsic factors and their mechanisms of action is essential for developing safe and effective interventions to manage allergic and inflammatory diseases [1,2,7]. Atopic dermatitis is characterized by a dysregulated immune response mediated by type 2 T-helper cells (Th2), and epigenetic modifications to immune-related genes can contribute to this immune dysregulation. The presence of genetic variations in immune-related genes that encode cytokines, including IL-4, IL-13, and IL-31, has been linked to a higher susceptibility to AD. Additionally, mutations in the filaggrin gene and other genes responsible for skin barrier function can also contribute to the disease [6]. Barrier dysfunction is another intrinsic factor in atopic dermatitis, with genetic mutations in skin barrier function genes, such as filaggrin, contributing to the disease [5]. Alterations in the skin microbiome, such as decreased microbial diversity and increased *Staphylococcus aureus* colonization, can also contribute to AD and can be influenced by both genetic and environmental factors [9]. (Figure 1)

## 3. Genetics of Atopic Dermatitis

Twin studies have illustrated the substantial influence of inter-individual variation in AD patients, with heritability estimates ranging from 69% to 80% [10]. The extended efforts of sequencing the human genome augmented the genetic epidemiology methods from genome-wide linkage analyses to the establishment of genetic association studies [11]. The latter has unveiled an abundance of genetic loci through direct sequencing focusing on the current understanding of the molecular pathophysiology of AD. In particular, candidate-gene studies have identified the genetic factors responsible for the impaired skin barrier, which is influenced by polymorphisms located within the Epidermal Differentiation Complex cluster (EDC). This cluster consists of more than fifty genes that encode proteins involved in the final differentiation and cornification of keratinocytes within the 1q21 chromosomal region [12,13]. Furthermore, the inflammatory aspect of AD has been associated with cytokines located within the Th2 cluster on the 5q31.1 locus [14]. Paradigms of AD risk loci include null variants mapped to the *FLG* gene, encoding the Filaggrin protein implicated in the formation of the stratum corneum, and the Th2-secreted IL-4 and IL-13 that serve as critical mediators of the allergic inflammation [15,16,17,18]. However, results from large-scale association studies and functional experiments support the increased role of the skin barrier in the disease predisposition and, thus, the underlying genetic variability [19]; variants mapped to the desmoglein family (e.g., DSG1 and DSG3) [20,21], corneodesmosin (CDSN) [22], and the protease inhibitor SPINK5 represent the epidermal barrier dysfunction as the primary pathogenetic event during eczema pathogenesis [23].

The advent of technological innovations led to the development of hypothesis-free genomic approaches through GWASs that scrutinize the entire genome for risk loci. GWAS findings have replicated significant findings regarding the *FLG*, *IL-13*, *OVOL1,* and *IL-6R* genes in European individuals [6,24,25]. AD pertains to a stable genetic background across diverse ethnicities, where 8/17 GWAS-significant loci in >2000 Japanese AD patients were shared with the European results [26]. All GWASs uncovered additional immune-related variants that constitute the central pathogenic axes of eczema. These inflammatory signals were further validated through the investigation of disease risk variants under the allergic march spectrum, incorporating AD, hay fever, and asthma. Ferreira and colleagues found 136 independent SNPs associated with allergy risk, where 130 variants showed equal contribution to all individual diseases [27]. Consequent assessment of the perturbed pathways through both SNP-based and gene-based approaches highlighted the implication of lymphocyte-mediated immunity in the allergic disease onset [28]. Gene prioritization approaches have also focused on AD following the integration of gene expression and genomic data. A transcriptome-wide association analysis (TWAS) reported 25 significant genes incorporating four novel protein-encoding loci (LINGO4, RFX5, P4HA2, RBM17). In contrast, enrichment analyses, including the total perturbed TWAS signals, displayed a tissue-specific enrichment pattern, mainly associated with cornified envelope formation in the skin and T cell-mediated immunity in peripheral blood [29]. Remarkably, unpublished GWAS meta-analysis that combines international collaborations with large biobank data further expanded the catalog of associated loci to 81 in European cases, however, explaining a small fraction of the overall heritability compared to twin studies, a finding supported by previously published GWAS [6].

One of the most profound applications of GWAS-derived data refers to the calculation of polygenic risk scores (PRSs), which summarize the impact of genetic variants in the disease predisposition to a single score, thereby providing an individualized framework for early risk assessment and preventive measures [30,31]. Genetic scores in AD have demonstrated the significant implication of exposure covariates in PRS calculations, including sex, age, and family history risk scores. Arehart and colleagues compared the predictive ability between AD-specific, leveraged allergy GWAS data as well as a combined model with the inclusion of *FLG* risk variants; predictably, the latter model reported the highest magnitude of strength and c-statistic, thus setting the framework for future implications of PRS computations for preventive medicine and early diagnosis [32]. Furthermore, systemic biomarker dysregulation was identified across different AD age groups. Proteomic analysis has revealed that beyond the common Th2 immune activation, the immune signatures of AD exhibit age-specific characteristics [33]. 

The declining cost of sequencing approaches has thus facilitated the employment of whole-genome sequencing (WGS) and whole-exome sequencing (WES) studies to facilitate the ‘missing heritability’ shortfall derived from the common genetic variants identified by GWAS. WES studies have governed the exploration for the rare variation in eczema with increasing sample sizes [34,35,36,37,38,39,40]; the largest WES study comprising ~20,000 AD cases documented 273 significant SNPs (*P*-value < 10–8) mapped to 11 loci with a minor allele frequency (MAF) < 0.001 [41]. Strikingly, rare (MAF < 0.01) and low-frequency (0.01 < MAF < 0.05) variants accounted for more than 20% of the SNP-based heritability of eczema, proving thus their role in the disease susceptibility [39]. Targeted sequencing approaches have also been employed to facilitate the identification of rare, functional variants in the genetic background of AD. Despite the contribution of loss-of-function *FLG* SNPs in the AD risk, machine-learning approaches revealed 48 low-frequency variants associated with increased self-reported remission after 6 months in 326 African American patients from the Pediatric Eczema Elective Registry (PEER) cohort in the absence of medications, 16 of which preserving the significant signals in an independent cohort [42]. The PEER cohort was utilized in additional studies reporting significant associations with rare variants mapped to *FLG2* and *TCHHL1* genes in both African American (*n* = 326) and white (*n* = 379) AD patients [43].

## 4. Epigenetics of Atopic Dermatitis

Despite the significant contribution of the inter-individual variability and heterogeneity in the etiology of the disease, the abundance of exposome related to AD dynamically affect the DNA sequence through reversible changes. Epigenetic modifications refer to alterations in the DNA sequence that orchestrate gene expression via modulating the accessibility of the transcriptional machinery through the addition of a methyl group to the C5 position of cytosine, resulting in the formation of 5-methylcytosine (5mC). On the contrary, demethylation of 5mC occurs through two processes, including ten-eleven translocation (TET) enzymes that form an intermediate product called 5-hydroxymethylcytosine (5hmC), and deamination of 5mC followed by the base excision repair (BER) pathway [44].

Targeted bisulfite sequencing has examined both promoter and intragenic regions of the *FLG* locus promoter regions of the *FLG* gene, with the former displaying the hypermethylation of a CpG (5′-C-phosphate-G-3′) region between lesional and non-lesional AD skin [45,46]. Furthermore, it has been illustrated that the combined effect of *FLG* variants and adjacent methylation status leads to an increased risk of developing the disease, although this finding was based on a small sample size. Relevant examples of targeted pyrosequencing include hypermethylated promoter regions of the skin-related HBD-1 and inflammation-related *NLRP2* genes, as well as hypomethylation of *TSLP* and *FCERG1G* genes, with the latter mediated by the *TSLP* over-expression in eczema individuals [47,48,49,50]. Notably, epigenetic modifications in the central Th2 commitment genes exhibit a predictive ability regarding eczema exacerbation during childhood; hypermethylation of the *IL-4R* CpG +28269 region in the umbilical cord was associated with the incidence of AD at 12 months and 6–7 years, suggesting putative novel biomarkers in disease onset and progression [51]. 

Epigenomic studies in AD patients have attempted to delineate the regulatory mechanisms that control the inflammatory response during disease maintenance. Nevertheless, DNA methylation profiling of circulating CD4+ T cells from AD patients failed to highlight any significant differentially methylated region (DMR) in 28 and 15 eczema cases [52,53]. Similarly, whole blood cells from 83 AD patients were also scrutinized for epigenetic alterations, showing in total six significant results contrary to 490 DMRs between healthy individuals and eczema herpeticum, implying thus the exploration of skin biopsies [54]. Indeed, the epigenomic profile of the epidermal tissue was significantly separated from whole blood biopsies, revealing, in addition, nine validated DMRs; transcriptional profiling of the same biopsies showed strong positive correlations between DNA methylation and gene expression, with most perturbed transcripts referring to the EDC and keratin clusters [52]. Subsequent analyses incorporating a larger number of CpG sites for evaluation confirmed the differential methylation of skin-related loci and cell proliferation [55]. Nonetheless, Acevedo and colleagues, through the investigation of circulating CD4+ CLA+ T cells, documented 49 DMRs mapped to regulatory regions of 35 genes compared to healthy individuals that orchestrate the inflammatory response during disease maintenance, with exemplars of *IL-10RA*, *IL-13* and *TOX2*. MiRNA profiling further elucidated the perturbed transcriptional mechanisms in AD, since several upregulated miRNAs targeted differentially methylated genes including *ESR1*, *NDFIP2*, *ASB2,* and *TNRC6A*. Clustering analysis of both omics’ approaches reported six distinct interconnected communities, with two clusters exhibiting the antagonistic behavior between the inflammation and homeostatic mechanisms during eczema exacerbation [56]. 

## 5. Noncoding RNAs in Atopic Dermatitis

Investigating post-transcriptional modifications presents an attractive approach to understanding the intrinsic molecular interactions that occur during disease development and maintenance, which are influenced by exposure to risk factors. Non-coding RNAs (ncRNAs), a diverse class of RNA molecules that do not encode for functional proteins, have emerged as critical regulators of the transcriptomics landscape of complex diseases with a key role in all cellular processes. The mechanism of action of ncRNAs incorporated a wide variety of post-transcriptional, epigenetic, and chromosomal modifications, with most studies focusing on miRNAs and lncRNAs.

Notwithstanding the importance of miRNAs, significant progress has been made towards the identification of novel RNA molecules with unique structures; circRNAs form a covalently closed loop structure lacking distinct, free ends that foster their prolonged half-lives compared to linear RNAs [57]. CircRNA molecules sequester miRNAs and display a sponge-like effect, as well as interact with RNA-binding proteins. Two studies have evaluated the expression profile of circRNAs in atopic dermatitis and psoriasis, depicting positive correlation signals between the lesional skin biopsies of both diseases. Moldovan and colleagues were the first to explore the expression of circRNAs in lesional AD biopsies through whole transcriptome sequencing, reporting a smaller perturbed profile in eczema patients compared to psoriasis individuals; however, most deregulated circRNAs in AD were shared between both cutaneous disorders [58]. Contrastingly, expression profiling of circRNAs in the peripheral blood of eczema patients provided the under-expression of a novel circRNA, *circ_0004287*; functional experiments reported its anti-inflammatory mechanisms through the repression of the *MALAT1* ncRNA, inhibiting the activation of M1 macrophages [59].

Various high-throughput and direct quantitative assays have been utilized to elucidate the mechanisms regulating inflammation in AD, with a particular focus on peripheral blood and related cells. Inconsistent findings were reported when comparing genome-wide miRNA levels in both serum and urine of 30 children with AD compared to 28 healthy individuals to those of lesional skin [60]. However, *miR-203* and *miR-483-3p* were identified as potential serum biomarkers for early detection of AD, with the downregulation of *miR-203* in urine samples [61]. *MiR-223* was found to be upregulated in patients’ whole blood cells and is involved in hematopoietic lineage differentiation processes and platelet activation, displaying a diverse expression profile in complex diseases [62,63]. In addition, central miRNAs associated with various inflammatory disorders, such as *miR-146a* and *miR-155*, were found to exhibit their primary mechanism of action in the pro-inflammatory NF-κB pathway [64,65,66,67,68]. Interestingly, the regulatory landscape of AD shares molecular similarities with several cancer types, with over-expression of *miR-151*, an oncogenic miRNA that facilitates metastasis, and repression of *miR-451a* and *miR-194-5p*, suggesting potential consensus mechanisms that could serve as early, identifiable risk factors in preventive medicine [69,70,71,72,73,74,75,76]. (Figure 2)

Despite the inherent differences and opposing underlying mechanisms between lesional skin and peripheral blood cells during inflammation, the pivotal regulators of inflammation, such as *miR-146a*, *mir-223,* and *miR-155,* persist in an upregulated profile in eczema biopsies [60,66,77,78,79,80]. However, Sonkoly et al. proved that skin-resident immune cells, particularly CD4+ CD3+ T cells, are responsible for the enhanced *mir-155* signals in AD skin biopsies through immunohistochemical staining [81]. In-depth analysis of publicly available transcriptomic datasets in eczema patients has revealed several regulatory interactions, competing endogenous RNA (ceRNA) networks that orchestrate the inflammatory response during AD pathogenesis and interactions between the expression profile of protein-coding and non-coding transcripts in AD biopsies with metabolic signatures [82,83,84,85]. Similarly to peripheral blood cells, the regulatory landscape of the cutaneous inflammation in AD shares a multitude of commonalities with the psoriasis inflammation, however, contradicting the discriminatory ability of serum miRNA levels between patients and healthy individuals [78]. Furthermore, targeted investigation of dysregulated miRNAs unveiled anti-inflammatory regulatory elements that could facilitate future therapeutic approaches. For instance, *mir-124*, a down-regulated molecule in AD lesional biopsies, was found to target the p65 subunit of the pro-inflammatory NF-κB pathway and subsequently reverse the cutaneous inflammation in TNF-stimulated keratinocytes [86]. Similarly, the IL-4-mediated STAT3 signaling could be repressed via the up-regulation of *mir-1294*, a tumor suppressor miRNA with significant participation in several cancer subtypes [87,88]. (Figure 2) Overall, studies on lesional skin have shown upregulation of *miR-10a*, *miR-24*, *miR-27a*, *miR-29b, miR-146a, miR-151a, miR-193a, miR-199, miR-211, miR-222, miR-4207,* and *miR-4529-3p* and downregulation of *miR-135a, miR-143, miR-184, miR-194-5p,* and *miR-4454* [89].

## 6. Regulatory Interactome in Atopic Dermatitis

Interactions between the relatively stable genetic background of complex diseases with the highly variable epigenomic and regulatory profile are intricate, displaying significant implications for disease development, progression, and treatment. The genomic variability can interfere with the transcriptional activity through variants mapped in regulatory regions and subsequently quantified by expression quantitative trait loci (eQTLs) and mediate the methylation status of specific loci by forming novel CpG sites [90].

Sobczyk et al. conducted an integrative analysis of 103 molecular data sets, including genomics, transcriptomics, and protein QTLs, to identify causal genes and pathways that contribute to eczema pathogenesis [91]. Their pipeline prioritized genes relevant to skin and peripheral blood expression signals for further exploration as potential pharmaceutical targets. Surprisingly, several established risk loci, such as *FLG*, *TSLP*, and *SPINK5*, were not prioritized, and the gene list showed only marginal enrichment for skin- and barrier-related terms, despite confirming the intricate inflammatory mechanisms underlying eczema pathogenesis targeted in therapeutic interventions involving IL-4, IL-13, and JAK-STAT signaling pathways. The study also provided new insights into tissue type-specific molecular interactions caused by causal variants, shedding light on previously unexplored aspects of eczema etiology.

Interactions between the genetic background of AD with epigenetic modifications are limited, however, furnishing valuable perspectives in the etiology of eczema. Recent studies have identified hypermethylation of the VSTM1 promoter region in monocytes, encoding the negative inflammatory regulator SIRL-1. This methylation profile is dependent on the allelic status of the VSTM1 rs612529T/C SNP, with the C allele indicating increased methylation and, thus, repressed expression. Significant insights were further provided for the independent KIF3A rs11740584 and rs2299007 risk variants, promoting the formation of CpG islands. Specifically, the rs11740584G and rs2299007G alleles form novel CpG sites that lead to the hypermethylation and suppression of the KIF3A expression, a gene responsible for the skin barrier dysfunction in Kif3αK14Δ/Δ mice, indicating a causal role in eczema pathogenesis through their interaction with epigenetic modifications [92].

## 7. Immune Dysregulation in Atopic Dermatitis

The immune system’s interaction with the skin is crucial in AD pathogenesis. As the body’s primary defense, the skin relies on a coordinated immune response to protect against infections. However, this balance is disrupted in eczema, leading to dysregulated immune responses that trigger chronic inflammation and compromise the skin’s protective barrier. The abnormal immune response in AD contributes to the complex nature of the disease, highlighting the need to understand the intricate immunological mechanisms for effective treatments. AD is influenced by a combination of inflammatory and environmental factors, resulting in persistent inflammation and an imbalanced immune response, primarily characterized by type 2 inflammation.

Moreover, inborn errors of immunity, typically associated with increased susceptibility to infections, can also lead to immune dysregulation, including allergic inflammation. Recently, the term primary atopic disorders (PADs) have emerged to describe heritable monogenic allergic disorders. Clinicians should be aware that allergic conditions such as AD, food allergy, and asthma can be manifestations of misdirected immunity in patients with inborn errors of immunity. The presence of severe, early-onset, or coexisting allergic conditions may signal an underlying PAD, and its early recognition is crucial for informed treatment decisions and improving patient outcomes. Next-generation sequencing can aid in the identification of monogenic allergic diseases, enabling precise therapeutic interventions targeting specific molecular defects [93,94,95,96].

The extrinsic exposome, such as allergens and mechanical injury, can trigger the release of pro-inflammatory cytokines, including thymic stromal lymphopoietin (TSLP), IL-33, IL-25, IL-18, and chemokines such as CCL17 and CCL22, from skin cells in the stratum corneum and dendritic cells resident in the skin. These factors create a hostile inflammatory environment that promotes increased sensitization [97,98,99,100,101,102]. IL-33 stimulation of group 2 innate lymphoid cells (ILC2s) causes the production of IL-5, while the polarization of CD4+ naïve T cells to the predominant Th2 phenotype causes secretion of IL-4, IL-5, and IL-13, which are the signature cytokines in AD. Therapeutic strategies target these cytokines as they play key roles in the pathogenesis of the disease [103,104,105]. The Th2-mediated cytokine cascade triggers molecular pathways with diverse mechanisms of action, including the pro-inflammatory JAK/STAT signaling pathway that affects immune cell differentiation and activation [106]. IL-13 is a key cytokine in the development of AD, with *miR-143* shown to be capable of regulating its receptor, IL-13Rα1, and preventing the IL-13-mediated dysregulation of proteins such as Filaggrin (FLG), Loricrin (LOR), and Involucrin (IVL), which are essential for the integrity of the skin barrier [107]. 

In addition, *miR-155-5p* has been shown to alter the expression of various proteins, including protein kinase inhibitor α, tight junction proteins such as occludin and claudins, and TSLP, thereby regulating allergic inflammation [108,109,110]. An increase of the *BIC* gene, which encodes the *miR-155* precursor, in the skin of AD patients compared to healthy controls, suggests that genetic variants in the *miR-155* gene may play a role in AD susceptibility; three of the five SNPs spanning the *BIC/miR-155* gene were associated with AD, however without statistical significance [111]. 

IL-13 binds to the IL-4Rα receptor, expressed on keratinocytes, fibroblasts, and immune cells, and is capable of inhibiting OVOL1 signaling as well as activating IL-24 production via periostin, with both pathways leading to suppression of *FLG* expression [112,113]. Notably, genes encoding the above molecules have all been identified as risk loci in the latest AD GWAS [6]. In contrast, IL-4 demonstrates its pathogenic role in AD by orchestrating the allergic response and is involved in the major histopathological features of AD through induction of immunoglobin E (IgE) production by B cells and suppression of antimicrobial peptides (AMPs) production in keratinocytes [114,115]. Recently IL-4Rα Q576R polymorphism was found to predispose to increased AD severity and aggravation of allergic skin inflammation in mice [116,117]. Furthermore, IgE secretion induces the degranulation of mast cells (MCs) and secretion of pro-inflammatory histamine, IL-31, and IL-6 [118,119,120], exacerbating itching and wounding and thus predisposing to microbial infections. The persistence of the described type 2 inflammation, which is supported by the co-regulatory contribution of additional signals such as Th1 and Th17 pathways, governs the progression of acute AD into a chronic inflammatory state [121,122,123]. 

## 8. Barrier Dysfunction in Atopic Dermatitis

Despite the significant contribution of immune dysregulation in the manifestation of AD, epidermal barrier dysfunction also holds a pivotal role. The skin barrier serves as a critical defense mechanism against extrinsic exposomes, including microbes, chemicals, and UV radiation. Comprising the stratum corneum, a layer of deceased keratinocytes and lipids, the skin barrier acts as a physical barrier to prevent the ingress of harmful substances. Additionally, it also plays a vital role in maintaining skin hydration by preventing water loss. However, in AD, there is a disturbance in the skin integrity, leading to increased water loss and increased susceptibility to irritants and allergens. This barrier dysfunction is considered a fundamental intrinsic disease factor involved in the development and progression of AD, and therefore unveiling the intricate interplay between immune dysregulation and barrier dysfunction is critical to elucidating the complex pathophysiology of AD.

The importance of the skin barrier in AD pathogenesis has been widely demonstrated through the consistent associations of *FLG* LOF variants with the disease predisposition. The dynamic process of keratinocyte (KC) migration from the basal cell layer towards the outermost layer of the skin, the stratum corneum, is accompanied by complex differentiation processes and the expression of distinct cellular markers [124]. Notably, the expression of filaggrin serves as an ideal marker during the final stages of keratinocyte differentiation, which occur in the granular and cornified layers. Proteolytic breakdown of pro-filaggrin into filaggrin monomers leads to the aggregation of keratin filaments, in conjunction with the expression of relevant structural proteins mapped to the EDC cluster (e.g., LOR, ILV, and SPRR proteins) [125]. These molecules are then crosslinked by transglutaminases (TGs), ultimately composing a scaffold-like structure to form the cornified envelope. At the uppermost layer of the skin, the keratin bundles foster the morphological transformation of KCs to form the corneocytes, flattened cells without intracellular organelles and nuclei [19]. Corneocytes are subsequently tightly interlinked with corneodesmosomes that are formed via the immobilization of desmosomes at the intercellular matrix mediated by TGs [126]. 

Filaggrin deficiency through LOF and structural variants significantly alters the structural composition of the cornified envelope, thus affecting the formation of corneocytes and enhancing the cutaneous sensitization to allergic factors [127,128,129,130]. Filaggrin deficiency can further exacerbate the inflammation and dysfunctional skin barrier through increasing pH, activating serine proteases (SPs) and kallikreins (*KLKs*) that participate in desquamation, a process that involves the constant replacement of corneocytes in the cornified envelope [131,132,133,134,135]. Variants mapped in both kallikrein-encoding genes (e.g., *KLK7*), as well as serine protease inhibitors (e.g., *SPINK5*), have been associated with eczema [23,136]. However, the implication of several exogenous factors, such as house dust mites (HDMs), allergens, and *S. aureus* infection with the exotoxins that induce type 2 inflammation through IgE and aggravate the epidermal barrier dysfunction, as well as the severe immunological background of the disease has established the inside-outside or outside-inside hypothesis debate in eczema pathogenesis, nevertheless suggested incorporating both factors and relevant molecular abnormalities [9,137].

In parallel, exposure to air pollutants induces oxidative stress in the skin, which has been demonstrated to compromise the integrity of the skin barrier by affecting transepidermal water loss (TEWL), initiating inflammatory responses, altering the pH of the stratum corneum, and impacting the skin microbiome [138,139]. Several oxidative stress markers in the stratum corneum of AD biopsies have exhibited a correlation with the severity of AD. The levels of carbonyl moieties, lipid peroxidation, and superoxide dismutase activity in skin biopsies of 75 patients with AD were evaluated and compared to diseased and normal controls. The findings revealed elevated levels of protein carbonyl moieties in AD, which were directly associated with the severity of the condition. Immunostaining with anti-DNP and anti-4-HNE antibodies indicated positive staining in the outermost layers of the stratum corneum, suggesting that environmental reactive oxygen species (ROS) might cause oxidation to proteins in the stratum corneum, and consequent barrier dysfunction and aggravation of AD [140].

## 9. Microbial Dysbiosis in Atopic Dermatitis

The profound diversity of microbial organisms that colonize the human body constitutes a captivating ecosystem, impacting human health and well-being through multifaceted interactions and mechanisms. In particular, the skin microbiome assumes a pivotal role in the host’s defense mechanisms, acting as an additional barrier against external pathogens, modulating immune responses, and contributing to the maintenance of cutaneous homeostasis [141]. Likewise, the gut microbiome participates in an abundance of physiological processes, such as metabolism, digestion, and immune responses, and has been implicated in systemic disorders; dysbiosis of microbial populations in both tissues can be perpetuated by aberrant immune responses, leading to a vicious cycle mediated by the inflammatory cascade [142,143]. In eczema, such interactions between the impaired skin barrier, perturbed immune response, and microbial alterations strongly participate in both the disease onset as well as the maintenance phase; nevertheless, molecular changes have yet to be systematically elucidated.

The perturbed microbial composition in patients with AD has been extensively characterized, with the presence of *S. aureus* governing the dysfunctional epithelial barrier along with the reduction of glycerol fermentation bacteria, such as *C. acnes* and *S. epidermis* [144,145]. Beheshti and colleagues conducted a comprehensive multi-omics assessment of saliva samples from 37 AD infants, employing targeted cytokine, human mRNA, miRNA, and 16s ribosomal RNA analyses to unveil putative novel pathological mechanisms in eczema onset [146]. Despite focusing on identified deregulated markers of AD, with the exemplars of *FLG*, *SPINK5*, *mir-146b,* and alpha diversity of Proteobacteria, significant differences were uncovered for several molecular factors, such as *mir-375*, *mir-21*, Th1/Th2 ratio (measured by the IL-8/IL-6 ratio) and Proteobacteria abundance, with the latter being positively correlated with the expression levels of *mir-375* (rho = 0.21). Alpha diversity also reported a positive correlation with the AD severity, further emphasizing the prominent role of the microbiome in disease pathogenesis. Notably, statistically significant factors from each -omics analysis showed a high discriminative ability as measured by the receiver operator characteristics curve (c-statistic = 0.814), providing the framework for additional investigation of such factors in the cutaneous inflammation. 

Despite the limited research on histone modification in the eczema landscape, Traisaeng and colleagues explored the potential therapeutic implication of butyric acid analogs in AD through the inhibition of *S. aureus* colonization and effects on histone modifications in HaCaT KCs [147]. Butyric acid, a short-chain fatty acid (SCFA), exhibits anti-microbial activity as well as represses the expression of histone deacetylase (HDAC), leading to increased transcriptional activity and has been proposed as a potent anti-inflammatory modulator [148,149]. The abundance of *S. aureus* in eczema skin was significantly reduced at in vitro co-culture with *S. epidermis*, accompanied by the presence of 2% glycerol through glycerol fermentation, as well as in AD mice suppressing the expression of IL-6. Administration of water-soluble butyric acid (BA–NH–NH–BA), the sole fermentation metabolite of *S. epidermidis*, in HaCaT KCs increased the levels of acetylated Histone H3 lysine 9 (AcH3K9), reporting as well dose-dependent reduction of cutaneous IL-6 levels. The investigation of histone modifications accompanied by the microbial diversity in eczema is of paramount importance to unveil the underlying molecular mechanisms and highlight possible therapeutic targets. 

## 10. Hormonal Effects and Pregnancy-Maternal Exposome

### 10.1. Hormonal Effects

AD is a skin disorder affected by hormones, as the skin itself is an endocrine organ [150]. Women are more likely to have AD than men, and hormonal changes during puberty, pregnancy, and menopause may trigger or worsen the disease [151]. In adolescence, the increase in sex hormones, such as estrogen and testosterone, is associated with changes in the immune system and skin barrier, which can influence the onset and severity of AD [152]. Studies on KFRS4/Kyo rats have shown that dermatitis with severe pruritus, which predominantly affects females, may be triggered by female sex hormones, such as estrogen or progesterone [153]. In the luteal phase of the menstrual cycle, there is a negative effect caused by a low estrogen/progesterone ratio that primarily affects the skin, impairing its barrier function and increasing its permeability. This, in turn, increases susceptibility to allergens and irritants. Additionally, during the premenstrual phase, increased levels of progesterone and estrogen can alter the activity of Th2 cells, exacerbating the symptoms of AD by promoting inflammation and IgE production [154,155]. Patients with intrinsic AD typically exhibit enhanced Th1 activity and a high incidence of nickel allergy, which also tends to be more prevalent in females [156,157]. Interestingly, skin biopsies from AD patients showed altered expression of two key regulators of steroidogenesis: the steroidogenic acute regulatory protein (StAR) and metastatic lymph node 64 (MLN64). StAR, which is critical for steroid synthesis, was absent in the basal layer of AD patient skin, while MLN64, a cholesterol transport protein, was reduced in the suprabasal layer [158]. Recently, IL-4 and IL-13 were found to promote androgen production and drive lipid abnormalities in sebocytes. The gene *HSD3B1*, which is known to produce the enzyme 3b-hydroxysteroid dehydrogenase 1 and play a role in sex hormone production, was unexpectedly found to be up to 60 times more active when exposed to IL-4 and IL-13, which are associated with AD and skin lipid production [159]. (Figure 2)

Estrogen has both pro- and anti-inflammatory effects on the immune system and can stimulate the production of inflammatory cytokines and increase the expression of adhesion molecules in the skin, leading to inflammation and itching. It can act on mast cells and induces IgE-mediated degranulation [160,161]. Increased during the mid-ovulatory cycle, serum estradiol and luteinizing hormone (LH) possibly influence the Th1/Th2 balance in AD, leading to a Th2 shift. 

Progesterone induces the secretion of TSLP expression and of progesterone-inducible blocking factor (PIBF), which in turn activates the Janus kinase 1 (Jak1)/signal transducer and activator of transcription 6 (STAT6) pathway; therefore, increases the production of Th2 cytokines such as IL-4 or IL-10 and modulates the function of immune cells towards a Th2 immune response [162,163,164]. Progesterone induces the dedifferentiation of Tregs but suppresses the differentiation of Th17 cells via suppressing the production of IL-17A, IL-17F, and IL-21, as well as the expression of RORc in human cord blood cells. Furthermore, progesterone can also suppress the phosphorylation of STAT3 in response to IL-6 [165]. Overall favors Th2/Treg activities but suppresses Th1/Th17 activities, which is particularly advantageous for the acceptance of an allogeneic fetus during pregnancy [166]. Interestingly, progestogen hypersensitivity, or autoimmune progesterone dermatitis (APD), is a rare hypersensitivity reaction associated with endogenous progesterone during the luteal phase and/or synthetic progestins [167].

Testosterone or dihydrotestosterone (DHT) has immunosuppressive effects on Th1, Th2, and Th17 activities, while it induces Treg activity and protects males against AD [168,169]. The adrenal cortex produces dehydroepiandrosterone (DHEA), which enhances Th1 responses, thus inhibiting the development of atopy [170]. Because females typically have higher levels of steroid sulfatase, an enzyme that converts dehydroepiandrosterone sulfate (DHEAS) to active DHEA, they may be more vulnerable to the effects of DHEA than males. Serum concentrations of DHEA or testosterone are lower in male AD patients compared to the reference group. Interestingly, prolonged physical stress with energy and sleep deprivation reduced serum DHEA levels and increased serum DHEAS levels. The decrease in DHEA levels could potentially account for the worsening of allergic diseases caused by stress [171,172].

Stress hormones such as cortisol and adrenaline can also impact AD symptoms, suppressing immune function and inflammation, but chronic stress can impair skin barrier function and exacerbate AD symptoms. Hormonal variations activate the hypothalamus-pituitary-adrenal axis and modulate skin neuropeptides, exacerbating AD symptoms during premenstrual hormonal variations [151].

During menopause, estrogen levels decrease, which can lead to changes in skin structure and function. Some studies have suggested that menopausal women with AD may experience more severe symptoms, such as increased pruritus and skin dryness. Hormone replacement therapy (HRT) may be an option to alleviate some of these symptoms in postmenopausal women with AD, but its effects on AD are still not well understood, and HRT use may be associated with other health risks. 

Although there is evidence of hormones influencing the expression of miRNAs, there is a limited amount of literature exploring this relationship in the context of atopic dermatitis (AD), necessitating further investigation. For instance, miR-223 has been found to be upregulated in AD, and its expression has been shown to be regulated by estrogen [173]. Similarly, in female skeletal muscle, postmenopausal hormone replacement therapy (HRT) has been found to decrease the expression of miR-182, miR-223, and miR-142-3p. This observed modulation of miRNAs has been linked to the enhanced expression of IGF-1R and FOXO3A, as well as the activation of insulin/IGF-1 pathway signaling through the phosphorylation of AKT and mTOR, which is an important mechanism for the positive impact of estrogen on the skeletal muscle of postmenopausal women [174]. Targeting pathways involved in the synthesis of sex steroid hormones may hold potential as a druggable approach to restore normal skin barrier in patients with AD.

### 10.2. Pregnancy-Maternal Exposome

Several maternal exposomal factors have been associated with a differentiated methylation profile in offspring, with exemplars of tobacco smoke affecting the methylation levels of *TSLP*, particulate matter 2.5 (PM2.5), and vitamin D levels in cord blood (CB) modulating the hypomethylation of *HLADRB1, DPP10,* and *AHRR*, as well as 25-hydroxyvitamin D (25[OH]D) deficiency, leading to the *MICAL3* hypomethylation and consequent mRNA upregulation [175,176,177,178]. Maternal anxiety, another associated prenatal risk factor for eczema, demonstrated patterns of an infiltration niche through the hypomethylation of the *MMP27* gene intra-linked with TNF signaling, belonging to the extended family of matrix metallopeptidases that participate during tissue remodeling and inflammatory diseases progression [179] (Figure 1).

Breastfeeding contains a wide range of beneficial ingredients, including biopolymers, which serve as a cross-link between the developing infant and the nurturing mother, strengthening the baby’s immune system and digestive health [180]. In relation to eczema, studies have shown that the expression of *mir-375-3p* increases during lactation and is associated with a lower risk of developing AD. Additionally, both *mir-375-3p* and *mir-148b-3p* are found in higher levels in healthy infants and are associated with the suppression of inflammation [181]. In infants with AD, *miR-144* expression was increased in cord serum at 1 year of age. This change was not seen in maternal serum or in serum at one year of age, and *miR-144* expression was not associated with total IgE levels at one year of age, suggesting that *miR-144* may play a role in the pathogenesis of AD independent of atopic status. The results showed that increased *miR-144* could activate the NF-kB pathway, leading to an increase in *hBD*-2 and *SERPINB4*, two molecules that have a proinflammatory role in AD [182] (Figure 2).

Similarly to epigenetic studies, miRNA abundance in CB has also been investigated in correlation with prenatal tobacco exposure; expression of two pathogenic miRNAs in maternal blood (MB) and CB were associated with elevated cotinine levels, the major metabolite of nicotine [183]. Remarkably, *mir-223* over-expression was associated with lower maternal as well as infant T regulatory (Treg) levels, thus provoking a dysregulated inflammatory response in children. Prenatal smoke exposure is also associated with an increased risk of AD in children, and miRNA abundance in cord blood has been investigated in correlation with prenatal tobacco exposure. *Mir-223* over-expression was associated with lower maternal as well as infant T regulatory (Treg) levels, resulting in a dysregulated inflammatory response in children [182].

Adductomics is a field of study that aims to identify all DNA adducts and the target sequence of each adduct, which can cause mutations and lead to cancer and birth defects. It is possible to measure adducts of electrophiles that result from reactions with DNA, glutathione, and blood proteins such as hemoglobin and human serum albumin [184]. Prenatal exposure to perfluoroalkyl and polyfluoroalkyl substances (PFASs) known as “forever chemicals,” such as perfluorooctane sulfonate (PFOS), was detected in 90% or more of pregnant women [2,185]. In utero exposure to PFOA has been associated with a higher risk of AD development as early as the age of 2 years old in children carrying GSTT1-null or GSTM1-null genotypes, which affect the glutathione S-transferase (GST) activity that is essential in chemical detoxification. GSTM1-null and GSTP1 Ile/Ile genotypes are also associated with an increased risk of AD in children with prenatal smoke exposure [186,187]. 

Environmental exposures can change the DNA methylation patterns of T helper cells. An epigenome-wide DNA methylation pattern examined more than 866,000 CpG sites in cord blood CD4+ T cells from children in a randomized allergy prevention trial. The study found that prenatal treatment with *Lactobacillus reuteri* and/or omega-3 fatty acids led to hypermethylation and affected immune- and allergy-related pathways in neonatal T helper cells [188]. Docosahexaenoic acid (DHA) supplementation during the second half of pregnancy had a small effect on infant DNA methylation [189]. Further studies are needed to determine whether these findings are related to AD development in children, as is the case with the Mediterranean Diet during Pregnancy study (PREMEDI) (clinical trial identified: NCT05119868) [190]. Understanding the role of DNA methylation in regulating the effects of perinatal interventions may be important in developing effective AD and allergy prevention strategies.

## 11. Conclusions

AD is a complex inflammatory cutaneous condition influenced by a combination of external and internal factors [191]. The exposome encompasses a person’s lifetime exposures, and specific periods such as prenatal development and adolescence are recognized as crucial stages in the development of AD. During these time points, the exposome can have enduring effects on the immune system, contributing to the manifestation of AD. Moreover, research is now focusing on the interactions between intrinsic pathways modulated by the extrinsic exposome, including the genome, the epigenome, the interactome, and factors, such as microbiome, immunity, hormones, and everyday lifestyle. Herein we adapted a multi-level conceptual framework for the exposome that embraces and recognizes the dynamic influence of the additive and interactive effect of the exposome alongside the genome.

## Data Availability

Not applicable.
